# Adherence to recommendations by infectious disease consultants and its influence on outcomes of intravenous antibiotic-treated hospitalized patients

**DOI:** 10.1186/1471-2334-12-292

**Published:** 2012-11-09

**Authors:** María-Carmen Fariñas, Gabriela Saravia, Jorge Calvo-Montes, Natividad Benito, Juan-José Martínez-Garde, Concepción Fariñas-Alvarez, Lorenzo Aguilar, Ramón Agüero, José-Antonio Amado, Luis Martínez-Martínez, Manuel Gómez-Fleitas

**Affiliations:** 1 Infectious Diseases Unit, Hospital Universitario Marques de Valdecilla, School of Medicine, University of Cantabria, Av. Valdecilla s/n, 39008 Santander, Spain; 2 Microbiology Department, Hospital Universitario Marques de Valdecilla, School of Medicine, University of Cantabria, Av. Valdecilla s/n, 39008, Santander, Spain; 3 Infectious Diseases Unit, Hospital Sant Pau, Sant Antoni Maria Claret, 167, 08025, Barcelona, Spain; 4 Pharmacy Department, Hospital Universitario Marques de Valdecilla, Av. Valdecilla s/n, 39008, Santander, Spain; 5 Preventive Medicine Department, Hospital Sierrallana, Bª Ganzo s/n, 39300, Torrelavega, Cantabria, Spain; 6 Microbiology Department, School of Medicine Universidad Complutense, Avda. Complutense s/n, 28040, Madrid, Spain; 7 Pneumology Department, Hospital Universitario Marques de Valdecilla, School of Medicine, University of Cantabria, Av. Valdecilla s/n, 39008, Santander, Spain; 8 Endocrinology Department, Hospital Universitario Marques de Valdecilla, School of Medicine, University of Cantabria, Av. Valdecilla s/n, 39008, Santander, Spain; 9 Department of Molecular Biology, University of Cantabria, Avda. Cardenal Herrera Oria s/n, 39011, Santander, Spain; 10 General Surgery Department, Hospital Universitario Marques de Valdecilla, School of Medicine, University of Cantabria, Av. Valdecilla s/n, 39008, Santander, Spain

**Keywords:** Infectious diseases specialists, Antibiotic intervention, Antibiotic use, Antibiotic management, Antimicrobial stewardship

## Abstract

**Background:**

Consultation to infectious diseases specialists (ID), although not always performed by treating physicians, is part of hospital’s daily practice. This study analyses adherence by treating physicians to written ID recommendations (inserted in clinical records) and its effect on outcome in hospitalized antibiotic-treated patients in a tertiary hospital in Spain.

**Methods:**

A prospective, randomized, one-year study was performed. Patients receiving intravenous antimicrobial therapy prescribed by treating physicians for 3 days were identified and randomised to intervention (insertion of written ID recommendations in clinical records) or non-intervention. Appropriateness of empirical treatments (by treating physicians) was classified as adequate, inadequate or unnecessary. In the intervention group, adherence to recommendations was classified as complete, partial or non-adherence.

**Results:**

A total of 1173 patients were included, 602 in the non-intervention and 571 in the intervention group [199 (34.9%) showing complete adherence, 141 (24.7%) partial adherence and 231 (40.5%) non-adherence to recommendations]. In the multivariate analysis for adherence (R^2^ Cox=0.065, p=0.009), non-adherence was associated with prolonged antibiotic prophylaxis (p=0.004; OR=0.37, 95%CI=0.19-0.72). In the multivariate analysis for clinical failure (R^2^ Cox=0.126, p<0.001), Charlson index (p<0.001; OR=1.19, 95%CI=1.10-1.28), malnutrition (p=0.006; OR=2.00, 95%CI=1.22-3.26), nosocomial infection (p<0.001; OR=4.12, 95%CI=2.27-7.48) and length of hospitalization (p<0.001; OR=1.01, 95%CI=1.01-1.02) were positively associated with failure, while complete adherence (p=0.001; OR=0.35, 95%CI=0.19-0.64) and adequate initial treatment (p=0.010; OR=0.39, 95%CI=0.19-0.80) were negatively associated.

**Conclusions:**

Adherence to ID recommendations by treating physicians was associated with favorable outcome, in turn associated with shortened length of hospitalization. This may have important health–economic benefits and stimulates further investigation.

**Trial registration:**

Current Controlled Trials ISRCTN83234896. http://www.controlled-trials.com/isrctn/sample_documentation.asp

## Background

Antibiotics account for one-third of hospital’s pharmacy budget, with between 25% and 50% hospitalized patients receiving antibiotics [1]. In hospitals, nearly all physicians prescribe antibiotics, and their prescriptions are influenced by characteristics of patient population, physician’s prescribing habits and local resistance patterns [2]. Inappropriate use of antibiotics results in a variety of adverse outcomes: narrow coverage increases the risk of therapeutic failure whereas broad coverage increases the risk of superinfection [3].

Antimicrobial stewardship by multidisciplinary teams [4] (including preventive medicine specialists, pharmacists, pharmacologists, microbiologists, infectious diseases specialists -IDs- and infection control nurses) is of increasing importance in the last decades due to the growing problem of nosocomial infections and resistance to antibiotics [5]. This together with the increasing proportion of hospitalized elderly patients with co-morbidities (that more frequently suffer infections) have contributed to the demand for more infectious disease services [6]. IDs can improve effectiveness by recommending a more appropriate antibiotic use [7], and their positive impact on patient care and infection control has been demonstrated [8,9]. The effectiveness of infectious disease consultations depends not only on clinically astute recommendations, but also on the adherence to them, since without it even the best recommendations are rendered ineffective [10]. Consultation to IDs, although not always performed by treating physicians, is part of hospital’s daily practice. The easy and rapid access to IDs has been identified as important factor facilitating consultation instead of seeking other sources of information, which are more time-consuming [7]. For this reason insertion of written ID recommendations in clinical records seems an strategy to be considered for rapid access to ID recommendations by treating physicians.

The aim of the present study was to investigate adherence by treating physicians to written ID recommendations inserted in clinical records and the potential influence that this adherence had on clinical outcome in hospitalized intravenous antibiotic-treated patients in a tertiary university teaching hospital in Spain.

### Patients and methods

A prospective, randomized, controlled study was carried out from January 2008 to December 2008 in the Hospital Universitario Marques de Valdecilla, Santander, Spain, a tertiary universitary hospital with 874 beds. The study protocol was approved by the Ethics Committee of the Autonomous Community of Cantabria (IFIMAV, Spain), the Fondo de Investigaciones Sanitarias [Registered number: FIS PI06/90094], and the Instituto de Formación e Investigación Marqués de Valdecilla (IFIMAV) [Registered number: API 06/03]. The patient’s informed consent was waived because the study was directed to treating physicians, not to patients, since the primary objective of the study was to assess adherence by treating physicians to ID recommendations using a new method described below. Potential influence of adherence by treating physicians on patient's outcomes was only secondarily assessed. The possibility of ID consultations is part of normal practice in the hospital, and as in routine practice, treating physicians participating in the study could ask for ID oral consultation at any time.

Patients admitted in General Surgery, Pneumology and Endocrinology departments that were receiving intravenous antimicrobial therapy prescribed by their responsible physicians for 3 days were identified by the Pharmacy department. The ID team was daily contacted and patients were randomised by groups (stratified randomization by clinical units) to intervention or non-intervention using the EPIDAT 3.1 programme (Dirección Xeral de Saúde Pública, Xunta de Galicia & Organización Panamericana de la Salud. Santiago de Compostela, Coruña, Spain, 2003). Interventions consisted of insertion in medical records of written treatment recommendations by an ID physician based on International [11-13] and Spanish [14-16] treatment guidelines adapted to local data on antimicrobial susceptibility after examination of patients, clinical records and microbiological data (if any) for each patient in the intervention group. Written recommendations (in specifically designed study forms) were inserted in the patient’s records in order to make it available to treating physicians, with a statement indicating availability for oral consultation if desired. Consultation was also available as part of daily practice for physicians treating patients in the non-intervention group (without insertion of written recommendations), but in the case of consultation the patient was withdrawn from the study. Paediatric patients, chronic dialysis patients, those receiving oral antibiotic therapy and those in which a member of the ID department had prescribed the initial antimicrobial regimen, were excluded.

Demographic and clinical data (including comorbidities), reason for antibiotic administration, regimen of antibiotic treatment, imaging and microbiological data, and microbiological and clinical outcomes were recorded both for patients in the intervention and non-intervention groups. The Charlson comorbidity index unadjusted by age [17] (age and sex were considered as separate variables), and the McCabe score [18] were calculated. Regimens of antibiotic treatment were classified as empirical, microbiologically-based or prolonged prophylaxis (antibiotic prophylaxis administered for >24 hours). Length of hospitalization (time from admission to discharge/death) was calculated. Patients with definite infection were those who met clinical criteria for infection with or without microbiological documentation. Infection was considered as nosocomial when signs/symptoms initiated 48-72h after admission and as healthcare-associated infection if infection occurred in patients who came from nursing homes or chronic care centres, had domiciliary hospitalization, had attended the hospital for oncological chemotherapy or had been hospitalized within the previous 30 days.

The appropriateness of empirical treatments prescribed by treating physicians (physicians of the primary service) was assessed and treatments were classified as adequate (agree with antibiotic choice, dosage, dosing interval and duration of treatment) or inadequate. Treatments were considered inadequate when one or several criteria following the Erbay’s modification [19] of Kunin’s criteria [20] were met. When IDs disagreed with the need for an antibiotic, treatments were classified as “unnecessary” by assigning a new category and thus eliminating this criterion from the Erbay’s category “inadequate”. In the intervention group, the degree of adherence to intervention was assessed at day +7 and +10 (from initiation of intravenous antibiotic treatment) and classified as complete (administration within 24h after consultation of the recommended antibiotic therapy with deviations lower than 20% for the dose and lower than ±30% for treatment duration), partial (administration of the recommended antibiotic therapy with deviations greater than 20% for the dose and/or greater than ±30% for duration and/or initiation between 24h and 48h after consultation) or non-adherence (non prescription of the recommended regimen or initiation after 48h from consultation). When the recommended duration of treatment was longer than 10 days, adherence was assessed at discharge.

Patients were followed during hospitalisation and at discharge they were clinically evaluated as “clinical success” (cure or improvement) or “clinical failure” (absence of improvement, worsening or death during hospitalization). Cure was considered when patients were discharged from the hospital with resolution of acute signs and symptoms of infection and improvement when patients were discharged from the hospital with oral antibiotic treatment. Patients with an initial positive culture and at least one follow-up culture were considered microbiologically evaluable and were evaluated as “microbiological success” (eradication) or “microbiological failure” (persistence or superinfection) at discharge.

Comparisons between proportions were performed by the χ2 test and the Fisher’s exact test, when necessary. For quantitative variables, since data did not showed normality in the Kolmogorov-Smirnoff test, the Kruskal-Wallis and Mann–Whitney tests, when necessary, were used. Bivariate analyses were performed comparing all variables between the group of patients with treatment showing adherence (complete adherence or complete plus partial adherence) versus those showing non-adherence, as well as between the group of patients showing clinical success versus those showing clinical failure. In order to avoid false associations in multiple comparisons in the bivariate analyses, the Bonferroni correction was applied and the p<0.001 was considered statistically significant to minimize type 1 error.Two different logistic regression models (step–wise procedure) were performed: one using “adherence” as dependent variable and another using “clinical failure”. In both multivariate analyses, independent variables were those showing differences (p≤0.1) in the previous bivariate analyses performed. In the multivariate analysis using “clinical failure” as dependent variable, adherence (both considered as complete adherence and as complete + partial adherence) was one of the independent variables introduced. Interactions and linear dependence between independent variables were previously controlled. Statistical analyses were performed using SPSS v 18 programme (SPSS Inc, Chicago IL). The models showing the highest R^2^ were considered.

## Results

### Disposition of patients

**Figure 1:**
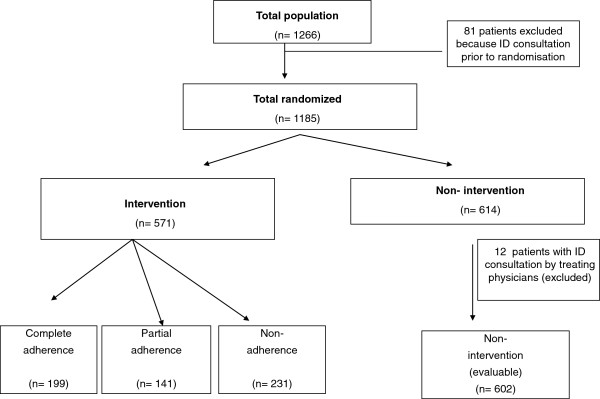
Study flow-chart.

A total of 1266 adult patients receiving intravenous antibiotic treatment for 3 days were identified by the Pharmacy department. Of them, 1185 patients were randomized and 1173 antibiotic-treated patients were finally considered, 571 patients in the intervention group and 602 patients in the non-intervention group. Figure 1 shows the study flow-chart including reasons for exclusion and withdrawns. Among the 571 patients in the intervention group, 199 (34.9%) showed complete adherence to the recommendation, 141 (24.7%) partial adherence and 231 (40.5%) non-adherence.

### Baseline demographic and clinical characteristics

Table 1 shows baseline demographic data, comorbidity and prognostic indices of patients included in the study distributed by study groups. Comorbidities present in >10% study patients were: previous surgery (62.0%), malignancies (41.2%), diabetes mellitus (39.7%), heart disease (38.5%), chronic respiratory insufficiency (32.9%), renal insufficiency (30.6%), immunosuppressive therapy (17.8%), smokers (14.1%) and alcohol intake (12.1%), without significant differences between the intervention and the non-intervention groups. The group of patients with intervention showed higher, although non-significant, Charlson index (p=0.006). When comparing groups by adherence to recommendations (complete versus partial versus non-adherence) significant differences were not found. The percentage of smokers (23.6% versus 13.4%, p=0.006) and of patients with chronic respiratory insufficiency (45.2% versus 32.9%, p=0.009) was non-significantly higher in the group of patients with complete adherence versus non-adherence. In the same way the percentage of patients with previous surgery was also non-significantly higher (44.2% versus 59.3%, p=0.002) in the non-adherence group.

**Table 1:** Baseline demographic and clinical characteristics for each group

	**Intervention**				**Non intervention**
	**Complete**	**Partial**	**Non adherence**	**Total**	
N	199	141	231	571	602
Age (years; mean ± SD)	64.9 ± 17.6	64.7 ± 15.8	64.8 ± 17.8	64.8 ± 17.2	65.0 ± 17.2
≤65	90 (45.2)	69 (48.9)	99 (42.8)	258 (45.2)	272 (45.2)
66-75	38 (19.1)	33 (23.4)	60 (26.0)	131 (22.9)	128 (21.3)
>75	71 (35.7)	39 (27.7)	72 (31.2)	182 (31.9)	202 (33.5)
Males	127 (63.8)	91 (64.5)	142 (61.5)	360 (63.0)	375 (62.3)
Coming from healthcare facilities	47 (23.6)	26 (18.4)	63 (27.3)	136 (23.8)	103 (17.1)
Prior hospitalization (previous 30 days)	56 (28.1)	41 (29.1)	76 (32.9)	173 (30.3)	150 (24.9)
Prior hospitalization (previous 90 days)	81 (40.7)	53 (37.6)	89 (38.5)	223 (39.1)	184 (30.6)
Ward of admission (Surgical)	102 (51.3)	89 (63.1)	158 (68.4) ^a^	349 (61.1)	423 (70.3)
Charlson (mean ± SD)	2.26 ± 2.04	2.74 ± 2.38	2.38 ± 2.15	2.43 ± 2.18	2. 09 ± 2.10
≤1	82 (41.2)	53 (37.6)	90 (39.0)	225 (39.4)	293 (48.7)
2	44 (22.1)	23 (16.3)	42 (18.2)	109 (19.1)	111 (18.4)
3	34 (17.1)	20 (14.2)	36 (15.6)	90 (15.8)	69 (11.5)
≥4	39 (19.6)	45 (31.9)	63 (27.3)	147 (25.7)	129 (21.4)
Mc Cabe (Fatal)	64 (32.2)	55 (39.0)	72 (31.2)	191 (33.4)	201 (33.4)
Reason for admission (infectious disease)	102 (51.3)	68 (48.2)	111 (48.1)	281 (49.2)	299 (49.7)

^a^p<0.001 versus complete adherence. Data expressed as n (%) except where indicated.

In relation to wards of admission, a significant higher percentage of patients admitted in surgical wards was found in the group with non-adherence (68.4% versus 51.3%, p<0.001).

Of the 1173 patients included, definitive infection was diagnosed in 913 (77.8%) patients, without differences in type and site of infections between groups. Community-acquired infections were the most frequent (597 out of 913, 65.4%) followed by nosocomial infections (277 out of 913, 30.3%). Healthcare associated infections only represented 4.3% (39 out of 913). By site of infection, lower respiratory tract infections were the most frequent (30.3%) followed by gastrointestinal infections (22.9%) and surgical site infections (22.1%).

### Baseline microbiological data

Microbiological tests were significantly (p<0.001) more frequently requested in the group of patients with complete adherence versus those with non-adherence (Table 2). Of the 747 initial cultures requested, 402 (53.8%) were positive, with non-significantly higher percentage of positive cultures (59.3% versus 48.1% p=0.002) in the intervention group. Overall, *Escherichia coli* (22.9%), *Streptococcus pneumoniae* (12.2%) and *Pseudomonas aeruginosa* (10.2%) were the most frequent species isolated.

**Table 2:** Microbiological tests [n (%)] and isolates (>10%) among those with positive cultures

	**Intervention**				**Non intervention**
	**Complete**	**Partial**	**Non adherence**	**Total**	
N	199	141	231	571	602
Patients with no microbiological tests	42 (21.1)	35 (24.8)	86 (37.2) ^a^	163 (28.5)	212 (35.2)
Patients with serology	17 (8.5)	3 (2.1)	9 (3.9)	29 (5.1)	22 (3.7)
Patients with culture	140 (70.4)	103 (73.1)	136 (58.8)	379 (66.4)	368 (61.1)
Positive cultures	80 (57.1)	65 (63.1)	80 (58.8)	225 (59.3)	177 (48.1)
*E. coli*	16 (20.0)	17 (26.2)	16 (20.0)	49 (21.8)	43 (24.3)
*S. pneumoniae*	13 (16.3)	6 (9.2)	9 (11.3)	28 (12.4)	21 (11.9)
*P. aeruginosa*	11 (13.8)	7 (10.8)	10 (12.5)	28 (12.4)	13 (7.3)

^a^p<0.001 versus complete adherence.

### Characteristics of antibiotic treatments prior to ID recommendations

Of the 1173 patients included, 209 (17.8%) patients received antibiotics as prolonged prophylaxis, with significantly higher percentage of patients receiving prophylaxis in the non-intervention group (Table 3). The percentage of initial treatments classified as adequate was significantly higher in the intervention versus non intervention group, in the complete versus partial adherence or versus non-adherence groups and in the partial adherence versus non-adherence group (Table 3). The most frequent reasons for classifying treatments as inadequate were inadequacy of treatment duration (48.8%) and lack of adequate dosage adjustment in patients with chronic renal or hepatic insufficiency (33.8%). Significant differences in the percentage of inadequate treatment duration were found between the intervention and non-intervention groups (38.4% versus 58.6%, p<0.001) and between complete adherence and non-adherence (16.1% versus 57.1%, p<0.001).

**Table 3:** Characteristics of antibiotic treatment prescribed before intervention [n (%)]

	**Intervention**				**Non intervention**
	**Complete**	**Partial**	**Non adherence**	**Total**	
N	199	141	231	571	602
Type of treatment					
Empirical	186 (93.5)	130 (92.2)	183 (79.2)	499 (87.4)	458 (76.1)
Prolonged Prophylaxis	13 (6.5)	10 (7.1)	45 (19.5) ^a^	68 (11.9)	141 (23.4) ^b^
Microbiologically-based	0 (0.0)	1 (0.7)	3 (1.3)	4 (0.7)	3 (0.5)
Antibiotic regimens including					
One compound	119 (59.8)	93 (66.0)	170 (73.6)	382 (66.9)	451 (74.9)
Two or more compounds	80 (40.2)	48 (34.0)	61 (26.4)	189 (33.1)	151 (25.1)
Treatment initiation in Emergency room	56 (28.1)	40 (28.4)	65 (28.1)	161 (28.2)	97 (16.1) ^b^
Treatment evaluation (initial)	199	141	231	571	602
Adequate	70 (35.2)	22 (15.6) ^a^	4 (1.7) ^a,c^	96 (16.8)	60 (10.0)
Inadequate	129 (64.8)	115 (81.6) ^a^	222 (96.1) ^a,c^	466 (81.6)	531 (88.2)
Unnecessary	0 (0.0)	4 (2.8)	5 (2.2)	9 (1.6)	11 (1.8)
Modification of initial treatment	129 (64.8)	52 (36.9) ^a^	33 (14.3) ^a,c^	214 (37.5)	105 (17.4) ^b^

^a^p<0.001 versus complete adherence; ^b^p<0.001 versus Total intervention; ^c^p<0.001 versus partial adherence.

Modifications of initial treatments were significantly (p<0.001) more frequent in the intervention group, and within this group, in the group of complete versus partial adherence or versus non-adherence and in the group of partial adherence versus non-adherence (Table 3).

### Outcome

Table 4 shows length of hospitalization and clinical and microbiological outcomes of patients included in the study distributed by study groups. Significantly (p<0.001) higher percentage of clinical success (cure + improvement) was found in the group of complete adherence (92.5%) versus non-adherence (80.1%) or versus the non-intervention group (81.9%). Mortality was 4.3%, without differences between groups. The percentage of patients showing microbiological eradication among the 190 microbiologically evaluable patients was significantly higher in the intervention group (41.9% versus 21.2%, p<0.001) and, within this group, in the group of complete adherence versus non-adherence (69.4% versus 20.0%, p<0.001).

**Table 4:** Clinical and microbiological outcome [n (%)] of patients included in the study

	**Intervention**				**Non intervention**
	**Complete**	**Partial**	**Non adherence**	**Total**	
N	199	141	231	571	602
Length of hospitalization (days; mean ±SD)	24.1 ± 29.3	26.5 ± 24.3	20.2 ± 18.8	23.1 ± 24.3	20.3 ± 33.8
**Clinical outcome**					
Cure + improvement	184 (92.5)	121 (85.8)	185 (80.1) ^a^	490 (85.8)	493 (81.9) ^a^
Death	4 (2.0)	5 (3.5)	12 (5.2)	21 (3.7)	30 (5.0)
**Microbiological outcome**					
No. patients microbiologically evaluable^b^	36 (18.1)	29 (20.6)	40 (17.3)	105 (18.4)	85 (14.1)
Eradication	25 (69.4)	11 (37.9)	8 (20.0) ^a^	44 (41.9)	18 (21.2) ^a,c^

^a^p<0.001 versus complete adherence; ^b^patients with initial positive culture and at least one follow-up culture; ^c^p<0.001 versus Total intervention.

### Factors associated with adherence and clinical outcome

The multivariate analysis using “adherence” as dependent variable was significant (R^2^ Cox=0.065, p=0.009), with adherence negatively associated with prolonged prophylaxis (p=0.004; OR=0.37, 95%CI=0.19-0.72).

Table 5 shows significant variables (p<0.1) in the bivariate analysis comparing patients showing success with those showing clinical failure. These variables were those included in the multivariate analysis using “clinical failure” as dependent variable and the results are shown in the Table. The model was significant (R^2^ Cox=0.126, p<0.001), with Charlson index (p<0.001; OR=1.19, 95%CI=1.10-1.28), malnutrition (p=0.006; OR=2.00, 95%CI=1.22-3.26), nosocomial infection (p<0.001; OR=4.12, 95%CI=2.27-7.48) and length of hospitalization (p<0.001; OR=1.01, 95%CI=1.01-1.02) associated with clinical failure. Complete adherence (p=0.001; OR=0.35, 95%CI=0.19-0.64) and adequate initial treatment (p=0.010; OR=0.39, 95%CI=0.19-0.80) were negatively associated with clinical failure.

**Table 5:** Factors predicting clinical failure

	**Success**	**Failure**	**P**	**OR**
N	983	190	Bivariate	Multivariate
Age (mean ± SD)	64.1 ± 17.6	68.9 ± 14.7	<0.001	p= 0.100
Coming from healthcare facilities	183 (18.6)	56 (29.5)	0.001	p= 0.215
Prior hospitalization (previous 30 days)	255 (25.9)	68 (35.8)	0.005	p= 0.625
Charlson index	2.07 ± 2.07	3.17 ± 2.16	<0.001	**1.19 (1.10-1.28) p<0.001**
Heart failure	358 (36.4)	94 (49.5)	0.001	p= 0.123
Renal insufficiency	281 (28.6)	86 (45.3)	<0.001	p= 0.155
Diabetes	408 (41.5)	93 (48.9)	0.058	p= 0.744
Malignancies	384 (39.0)	104 (54.7)	<0.001	p= 0.775
Immunosuppressive therapy	178 (18.1)	47 (24.7)	0.034	p= 0.262
Malnutrition	74 (7.5)	34 (17.9)	<0.001	**2.00 (1.22-3.26) p=0.006**
Nosocomial infection	187 (25.3)	88 (51.8)	<0.001	**4.12 (2.27-7.48) p<0.001**
Microbiological tests	652 (66.3)	160 (84.2)	<0.001	p= 0.020
Complete adherence	184 (18.7)	15 (7.9)	0.001	**0.35 (0.19-0.64) p=0.001**
Treatment initiated in Emergency room	777 (79.0)	138 (72.6)	0.051	p= 0.023
Adequate initial treatment	147 (15.0)	9 (4.7)	<0.001	**0.39 (0.19-0.80) p=0.010**
Modification of initial treatment	306 (31.1)	43 (22.6)	0.019	p= 0.140
Length of hospitalization	18.9 (27.0)	36.3 (37.4)	<0.001	**1.01 (1.01-1.02) p<0.001**

It shows only significant variables in the bivariate analysis. “Clinical failure” was used as dependent variable.

When the multivariate analysis was performed including complete plus partial adherence as independent variable (instead of complete adherence), the multivariate analysis was also significant (identical R^2^ Cox) with the same variables positively or negatively associated with clinical failure. In the group of patients showing clinical success, complete + partial adherence was 31.0% while it was 18.4% among patients showing clinical failure. Complete + partial adherence was negatively associated with clinical failure (p<0.001; OR=0.43, 95%CI=0.28-0.67).

## Discussion

Most ID specialists believe that it is evident that their care is valuable, although there are few published studies on how frequently their recommendations are followed, or clinical consequences of following them [10,21]. Antimicrobial stewardship advocates the use of the most suitable antibiotic in the context of the presenting clinical condition and specific patient, and its success rely on coordination and collaboration between healthcare professionals to ensure consistency in approach, shared knowledge and widespread diffusion of practice [4]. Among strategies that have been used to decrease injudicious antimicrobial use, streamlining involves expert review of patient’s antimicrobial regimens making recommendations to their providers about stopping or narrowing therapy [3]. However streamlining is applied after antimicrobial therapies have been initiated, allowing some degree of inappropriate exposure in addition to the potential limitation of effectiveness derived from the voluntary nature of its compliance [3,22].

There is uncertainty about factors affecting adherence to recommendations [10]. In the present study, adherence (complete plus partial) was 59.5% or even lower if we consider only patients with complete adherence (34.9%). Although the percentage of complete + partial adherence is within the frame of 53% to 90% reported in the literature [10,23], complete adherence was lower. This low adherence in our study is worrying and can be related to the fact that eligible patients were identified when they were receiving antibiotic treatment for 3 days, a fact favoring not to follow recommendations in those patients with early favorable responses. In the multivariate analysis only prolonged prophylaxis was significantly associated with non-adherence. This contrast with the idea of expecting more requests from surgical units since they are less experienced with infectious diseases [7].

Although the impact of infectious diseases consultations on actual patient management is difficult to assess [6], several studies have shown that consultations optimize antibiotic use in patients receiving intravenous antimicrobials and are cost-effective [24,25]. In the present study the group of patients with complete adherence to recommendations showed significant higher clinical success and eradication among microbiologically evaluable patients. Not surprisingly, Charlson index, malnutrition and nosocomial infections were the variables significantly associated with clinical failure in the multivariate analysis, but more importantly, adequate initial treatment and adherence to recommendations were negatively associated. These last factors are those that could be influenced by IDs in daily practice. In addition, microbiological tests were more frequently requested in patients randomized to intervention, with higher percentage of positive cultures, suggesting that ID consultation in addition to antibiotic advice also consciously or unconsciously influenced test request practices, and possibly led to more appropriate selection of sites for microbiological tests. These findings in patients receiving intravenous antibiotic therapy show that ID consultations not only improve patient’s outcome but may also result in substantial cost reductions since clinical failure was significantly associated with larger length of hospitalization. Decreasing the hospital length of stay by promoting earlier hospital discharge significantly reduce overall costs and increase the efficiency and cost-effectiveness of the hospital [26].

Several study limitations should be taken into consideration when extrapolating results to other settings since this study was performed in a single hospital, with participation of reduced number of specific wards, and only considering the subset of hospitalized patients receiving at least 3 days of intravenous antibiotic therapy. Probably local medical routines (differences in IDs daily practice) could influence extrapolations to other settings.

In conclusion, the method used for intervention (written recommendations inserted in clinical records 3 days after initiation of intravenous therapy) did not achieved a high degree of adherence despite this method made recommendations easily available to treating physicians. It has been reported that direct, personal communication [27] or the presence at patient’s bedside of ID specialists [28] would dramatically improved adherence to recommendations, although this is not always possible in daily practice. Regardless the low adherence in the present study, the results showed that adherence to recommendations were associated with favorable clinical and microbiological outcomes and shortened length of hospitalization. The results of the present study stress the importance of adherence (without it even the best recommendations are rendered ineffective) [10] and suggest important health–economic benefits. This stimulates further investigation in this field, with different methodologies and specific groups of patients, since as has been indicated, the need for physician expertise and intervention has never been more apparent than at the present time [29].

## Abbreviation

IDs: Infectious Diseases specialists.

## Competing interests

G. Saravia has received an educational grant from the Instituto de Formación e Investigación Marqués de Valdecilla (IFIMAV) for this study. All other authors: none to declare.

## Authors’ contributions

Conceived and designed the study: M-CF, NB, CF-A, RA, J-AA, LM-M, MG-F. Collection of data: GS, JC-M, J-JM-G. Analyzed the data: M-CF, CF-A, LA. Wrote the paper: M-CF, LA. Reviewed and approved the manuscript: M-CF, GS, JC-M, NB, J-JM-G, CF-A, LA, RA, J-AA, LM-M, MG-F.

## Pre-publication history

The pre-publication history for this paper can be accessed here:

http://www.biomedcentral.com/1471-2334/12/292/prepub

## References

[B1] Ansari F Gray K Nathwani D Phillips G Ogston S Ramsay C Davey P Outcomes of an intervention to improve hospital antibiotic prescribing: interrupted time series with segmented regression analysis J Antimicrob Chemother 2003 52 842 848 10.1093/jac/dkg459 14563900

[B2] Saizy-Callaert S Causse R Furhman C Le Paih MF Thébault A Chouaïd C Impact of a multidisciplinary approach to the control of antibiotic prescription in a general hospital J Hosp Infect 2003 53 177 182 10.1053/jhin.2002.1307 12623317

[B3] Gross R Morgan AS Kinky DE Weiner M Gibson GA Fishman NO Impact of a hospital-based antimicrobial management program on clinical and economic outcomes Clin Infect Dis 2001 33 289 295 10.1086/321880 11438891

[B4] Charani E Cooke J Holmes A Antibiotic stewardship programmes—what’s missing? J Antimicrob Chemother 2010 65 2275 2277 10.1093/jac/dkq357 20851812

[B5] Owens RC Jr Antimicrobial stewardship: concepts and strategies in the 21st century Diagn Microbiol Infect Dis 2008 61 110 128 10.1016/j.diagmicrobio.2008.02.012 18384997

[B6] Schlesinger Y Paltiel O Yinnon AM Analysis and impact of infectious disease consultations in a general hospital J Hosp Infect 1998 40 39 46 10.1016/S0195-6701(98)90023-8 9777520

[B7] Pavese P Sellier E Laborde L Gennai S Stahl JP François P Requesting physicians' experiences regarding infectious disease consultations BMC Infect Dis 2011 11 62 10.1186/1471-2334-11-62 21401916 PMC3061908

[B8] Sellier E Pavese P Gennai S Stahl JP Labarère J François P Factors and outcomes associated with physicians' adherence to recommendations of infectious disease consultations for inpatients J Antimicrob Chemother 2010 65 156 162 10.1093/jac/dkp406 19910328

[B9] Nagao M Iinuma Y Saito T Matsumura Y Shirano M Matsushima A Takakura S Ito Y Ichiyama S Close cooperation between infectious disease physicians and attending physicians can result in better management and outcome for patients with Staphylococcus aureus bacteraemia Clin Microbiol Infect 2010 16 1783 1788 10.1111/j.1469-0691.2010.03156.x 21077985

[B10] Lo E Rezai K Evans AT Madariaga MG Phillips M Brobbey W Schwartz DN Wang Y Weinstein RA Trenholme GM Why don't they listen? Adherence to recommendations of infectious disease consultations Clin Infect Dis 2004 38 1212 1218 10.1086/383315 15127330

[B11] Solomkin JS Mazuski JE Baron EJ Sawyer RG Nathens AB DiPiro JT Buchman T Dellinger EP Jernigan J Gorbach S Chow AW Bartlett J Infectious Diseases Society of America. Guidelines for the selection of anti-infective agents for complicated intra-abdominal infections Clin Infect Dis 2003 37 997 1005 10.1086/378702 14523762

[B12] Stevens DL Bisno AL Chambers HF Everett ED Dellinger P Goldstein EJ Gorbach SL Hirschmann JV Kaplan EL Montoya JG Wade JC Infectious Diseases Society of America. Practice guidelines for the diagnosis and management of skin and soft-tissue infections Clin Infect Dis 2005 41 1373 1406 10.1086/497143 16231249

[B13] Mandell LA Wunderink RG Anzueto A Bartlett JG Campbell GD Dean NC Dowell SF File TM Jr Musher DM Niederman MS Torres A Whitney CG Infectious Diseases Society of America; American Thoracic Society. Infectious Diseases Society of America/American Thoracic Society consensus guidelines on the management of community-acquired pneumonia in adults Clin Infect Dis 2007 44 2 S27 S72 10.1086/511159 17278083 PMC7107997

[B14] Tellado JM Sitges-Serra A Barcenilla F Palomar M Serrano R Barberán J Moya M Martínez M García-Rodríguez JA Mensa J Prieto J Guidelines for the empirical antibiotic treatment of intraabdominal infections [Article in Spanish] Rev Esp Quimioter 2005 18 179 186 16130041

[B15] Cisneros-Herreros JM Cobo-Reinoso J Pujol-Rojo M Rodríguez-Baño J Salavert-Lletí M Guidelines for the diagnosis and treatment of patients with bacteriemia. Guidelines of the Sociedad Española de Enfermedades Infecciosas y Microbiología Clínica [Article in Spanish] Enferm Infecc Microbiol Clin 2007 25 111 130 10.1016/S0213-005X(07)74242-8 17288909

[B16] Alvarez F Bouza E García-Rodríguez JA Mensa J Picazo JJ Sobradillo V Torres A Moya Mir M Pérez Escanilla F Puente T Cañada JL Martínez Ortiz De Zárate M Second consensus statement on the use of antimicrobial drugs in chronic obstructive pulmonary disease exacerbations [Article in Spanish] Rev Esp Quimioter 2002 15 375 385 12607552

[B17] Charlson ME Pompei P Ales KL MacKenzie CR A new method of classifying prognostic comorbidity in longitudinal studies: development and validation J Chronic Dis 1987 40 373 383 10.1016/0021-9681(87)90171-8 3558716

[B18] Mc Cabe WR Jackson GG Gram-negative bacteremia. II. Clinical, laboratory and therapeutic observations Arch Intern Med 1962 110 856 864 10.1001/archinte.1962.03620240038007

[B19] Erbay A Bodur H Akıncı E Çolpan A Evaluation of antibiotic use in intensive care units of a tertiary care hospital in Turkey J Hosp Infect 2005 59 53 61 10.1016/j.jhin.2004.07.026 15571854

[B20] Kunin CM Tupasi T Craig WA Use of antibiotics. A brief exposition of the problem and some tentative solutions Ann Intern Med 1973 79 555 560 4795880 10.7326/0003-4819-79-4-555

[B21] Petrak RM Sexton DJ Butera ML Tenenbaum MJ MacGregor MC Schmidt ME Joseph WP Kemmerly SA Dougherty MJ Bakken JS Curfman MF Martinelli LP Gainer RB The value of an infectious diseases specialist Clin Infect Dis 2003 36 1013 1017 10.1086/374245 12684914

[B22] Fraser GL Stogsdill P Dickens JD Jr Wennberg DE Smith RP Jr Prato BS Antibiotic optimization. An evaluation of patient safety and economic outcomes Arch Intern Med 1997 157 1689 1694 10.1001/archinte.1997.00440360105012 9250230

[B23] Sellier E Labarère J Gennai S Bal G François P Pavese P Compliance with recommendations and clinical outcomes for formal and informal infectious disease specialist consultations Eur J Clin Microbiol Infect Dis 2011 30 887 894 10.1007/s10096-011-1172-7 21311942

[B24] Lemmen SW Becker G Frank U Daschner FD Influence of an infectious disease consulting service on quality and costs of antibiotic prescriptions in a university hospital Scand J Infect Dis 2001 33 219 221 10.1080/00365540151060923 11303814

[B25] Gums JG Yancey RW Jr Hamilton CA Kubilis PS A randomized, prospective study measuring outcomes after antibiotic therapy intervention by a multidisciplinary consult team Pharmacotherapy 1999 19 1369 1377 10.1592/phco.19.18.1369.30898 10600085

[B26] Nathwani D Impact of methicillin-resistant Staphylococcus aureus infections on key health economic outcomes: does reducing the length of hospital stay matter J Antimicrob Chemother 2003 51 2 ii37 ii44 12730141 10.1093/jac/dkg250

[B27] Tenenbaum MJ Infectious diseases consultative recommendations: If heard, they can be listened to Clin Infect Dis 2004 38 1219 1221 10.1086/383327 15127331

[B28] Méan M Pavese P Tudela E Dinh-Van KA Mallaret MR Stahl JP Consultations with infectious disease specialists for patients in a teaching hospital: Adherence in 174 cases [Article in French] Presse Med 2006 35 1461 1466 10.1016/S0755-4982(06)74835-7 17028534

[B29] McQuillen DP Petrak RM Wasserman RB Nahass RG Scull JA Martinelli LP The value of infectious diseases specialists: non-patient care activities Clin Infect Dis 2008 47 1051 1063 10.1086/592067 18781883

